# The genome sequence of the Tufted Button, *Acleris cristana *(Denis & Schiffermüller, 1775)

**DOI:** 10.12688/wellcomeopenres.19508.1

**Published:** 2023-06-07

**Authors:** Douglas Boyes, Clare Boyes, University of Oxford and Wytham Woods Genome Acquisition Lab, Darwin Tree of Life Barcoding collective, Wellcome Sanger Institute Tree of Life programme, Wellcome Sanger Institute Scientific Operations: DNA Pipelines collective, Tree of Life Core Informatics collective, Darwin Tree of Life Consortium

**Affiliations:** 1 UK Centre for Ecology & Hydrology, Wallingford, England, UK; 2 Independent researcher, Welshpool, Wales, UK

**Keywords:** Acleris cristana, tufted button, genome sequence, chromosomal, Lepidoptera

## Abstract

We present a genome assembly from an individual female *Acleris cristana* (the Tufted Button; Arthropoda; Insecta; Lepidoptera; Tortricidae). The genome sequence is 562.6 megabases in span. Most of the assembly is scaffolded into 31 chromosomal pseudomolecules, including the W and Z sex chromosomes. The mitochondrial genome has also been assembled and is 16.1 kilobases in length. Gene annotation of this assembly on Ensembl identified 12,598 protein coding genes.

## Species taxonomy

Eukaryota; Opisthokonta; Metazoa; Eumetazoa; Bilateria; Protostomia; Ecdysozoa; Panarthropoda; Arthropoda; Mandibulata; Pancrustacea; Hexapoda; Insecta; Dicondylia; Pterygota; Neoptera; Endopterygota; Amphiesmenoptera; Lepidoptera; Glossata; Neolepidoptera; Heteroneura; Ditrysia; Apoditrysia; Tortricoidea; Tortricidae; Tortricinae; Tortricini; *Acleris*; *Acleris cristana* (Denis & Schiffermüller, 1775) (NCBI:txid758705).

## Background

*Acleris cristana* (the Tufted Button) is a micro-moth in the family Tortricidae. The species has a southerly distribution in Britain and is found throughout mainland Europe. There are also a few records from Japan ( GBIF Secretariat, 2023).

*A. cristana* is probably the most variable species amongst British Lepidoptera with over 130 named forms ( Sterling & Parsons, 2018). It is thought that the several of the genes influencing colours of individual pattern elements found on the wings of *A. cristana* segregate independently, which has resulted in numerous forms with very little gradation between them. In contrast, the closely related species, *A. hastiana*, which is also very variable, demonstrates many intermediate forms making it difficult to separate out the named forms ( Hancock *et al.,* 2015). In *A. cristana*, although the forewing colour varies significantly, the moth almost always has a distinctive tuft of raised scales in the centre of the forewing, giving rise to the common name of ‘Tufted Button’. 

The adult moth flies at dusk and in the UK is on the wing from September to mid-April. However, this includes a period of dormancy, from late autumn until early spring, after which the moth awakens to mate. Eggs are laid singly, or in small groups, on twigs of trees in the Rosaceae family, usually blackthorn ( *Prunus spinosa*). The larvae can be found in rolled leaf edges, and later instars are found in spun leaves. The larvae pupate between June to August either in folded leaves, or on the ground in leaf litter ( Emmet, 2010). The moth occasionally comes to light but can also be found by beating shrubs in the day.

A genome sequence from *A. cristana* will be useful for research into colour variation in moths, and more generally for comparative studies across the Lepidoptera. The genome of *A. cristana* was sequenced as part of the Darwin Tree of Life Project, a collaborative effort to sequence all named eukaryotic species in the Atlantic Archipelago of Britain and Ireland. Here we present a chromosomally complete genome sequence for *A. cristana* based on one female specimen from Wytham Woods, Oxfordshire, UK.

## Genome sequence report

**Figure 1.**
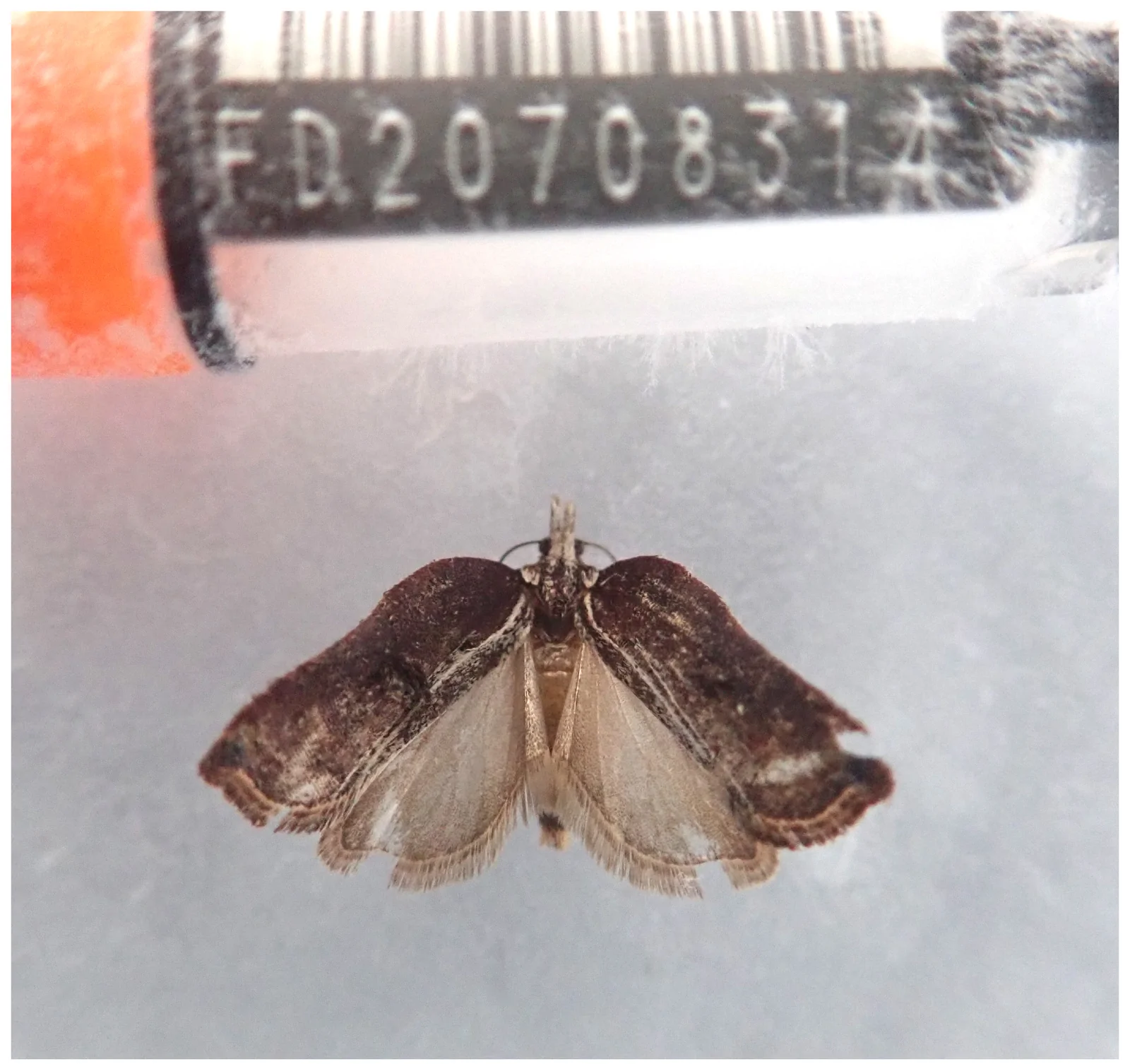
Photograph of the *Acleris cristana* (ilAclCris2) specimen used for genome sequencing.

The genome was sequenced from one female *Acleris cristana* ( Figure 1) collected from Wytham Woods, Oxfordshire, UK (51.77, –1.34). A total of 37-fold coverage in Pacific Biosciences single-molecule HiFi long reads was generated. Primary assembly contigs were scaffolded with chromosome conformation Hi-C data. Manual assembly curation corrected 53 missing joins or mis-joins and removed six haplotypic duplications, reducing the assembly length by 0.4% and the scaffold number by 36.62%, and increasing the scaffold N50 by 10.95%.

**Figure 2.**
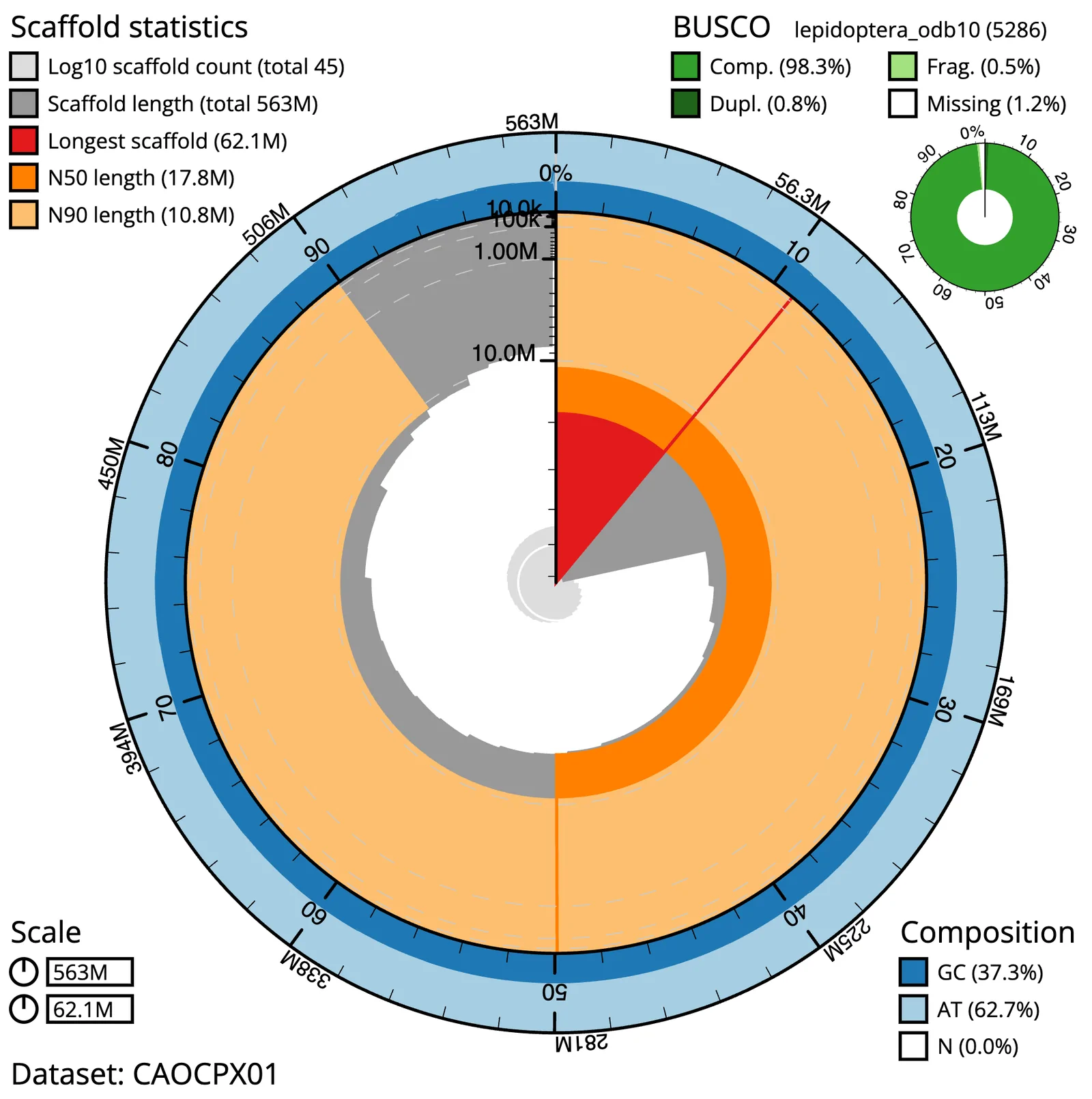
Genome assembly of *Acleris cristana*, ilAclCris2.1: metrics. The BlobToolKit Snailplot shows N50 metrics and BUSCO gene completeness. The main plot is divided into 1,000 size-ordered bins around the circumference with each bin representing 0.1% of the 562,572,194 bp assembly. The distribution of scaffold lengths is shown in dark grey with the plot radius scaled to the longest scaffold present in the assembly (62,083,584 bp, shown in red). Orange and pale-orange arcs show the N50 and N90 scaffold lengths (17,811,303 and 10,842,494 bp), respectively. The pale grey spiral shows the cumulative scaffold count on a log scale with white scale lines showing successive orders of magnitude. The blue and pale-blue area around the outside of the plot shows the distribution of GC, AT and N percentages in the same bins as the inner plot. A summary of complete, fragmented, duplicated and missing BUSCO genes in the lepidoptera_odb10 set is shown in the top right. An interactive version of this figure is available at https://blobtoolkit.genomehubs.org/view/ilAclCris2.1/dataset/CAOCPX01/snail.

The final assembly has a total length of 562.6 Mb in 45 sequence scaffolds with a scaffold N50 of 17.8 Mb ( Table 1). Most (99.72%) of the assembly sequence was assigned to 31 chromosomal-level scaffolds, representing 29 autosomes and the Z and W sex chromosomes. The W and Z chromosomes are similar in size, and are large chromosomes, in keeping with the cytogenetic findings of ( Šíchová *et al.*, 2013). The Z chromosome was identified based on alignment with *Acleris emargana* (GCA_927399475.2) which was assembled from a male sample (Z chromosome only). Chromosome-scale scaffolds confirmed by the Hi-C data are named in order of size ( Figure 2– Figure 5; Table 2). While not fully phased, the assembly deposited is of one haplotype. Contigs corresponding to the second haplotype have also been deposited. The mitochondrial genome was also assembled and can be found as a contig within the multifasta file of the genome submission.

**Table 1.** Genome data for *Acleris cristana*, ilAclCris2.1.

Project accession data		
Assembly identifier	ilAclCris2.1	
Species	*Acleris cristana*	
Specimen	ilAclCris2	
NCBI taxonomy ID	758705	
BioProject	PRJEB58659	
BioSample ID	SAMEA8603216	
Isolate information	ilAclCris2, female; whole organism (PacBio sequencing) ilAclCris1; whole organism (Hi-C scaffolding)	
Assembly metrics *		*Benchmark*
Consensus quality (QV)	65.9	*≥ 50*
*k*-mer completeness	100%	*≥ 95%*
BUSCO **	C:98.3%[S:97.6%,D:0.8%], F:0.5%,M:1.2%,n:5,286	*C ≥ 95%*
Percentage of assembly mapped to chromosomes	99.72%	*≥ 95%*
Sex chromosomes	Z and W chromosomes	*localised homologous pairs*
Organelles	Mitochondrial genome assembled	*complete single alleles*
Raw data accessions		
PacificBiosciences SEQUEL II	ERR10753928	
Hi-C Illumina	ERR10742410	
Genome assembly		
Assembly accession	GCA_948252455.1	
*Accession of alternate haplotype*	GCA_948250105.1	
Span (Mb)	562.6	
Number of contigs	156	
Contig N50 length (Mb)	6.5	
Number of scaffolds	45	
Scaffold N50 length (Mb)	17.8	
Longest scaffold (Mb)	62.1	
Genome annotation		
Number of protein-coding genes	12,598	
Number of non-coding genes	1,631	
Number of gene transcripts	22,144	

* Assembly metric benchmarks are adapted from column VGP-2020 of “Table 1: Proposed standards and metrics for defining genome assembly quality” from ( Rhie *et al.*, 2021). ** BUSCO scores based on the lepidoptera_odb10 BUSCO set using v5.3.2. C = complete [S = single copy, D = duplicated], F = fragmented, M = missing, n = number of orthologues in comparison. A full set of BUSCO scores is available at https://blobtoolkit.genomehubs.org/view/ilAclCris2.1/dataset/CAOCPX01/busco.

**Figure 3.**
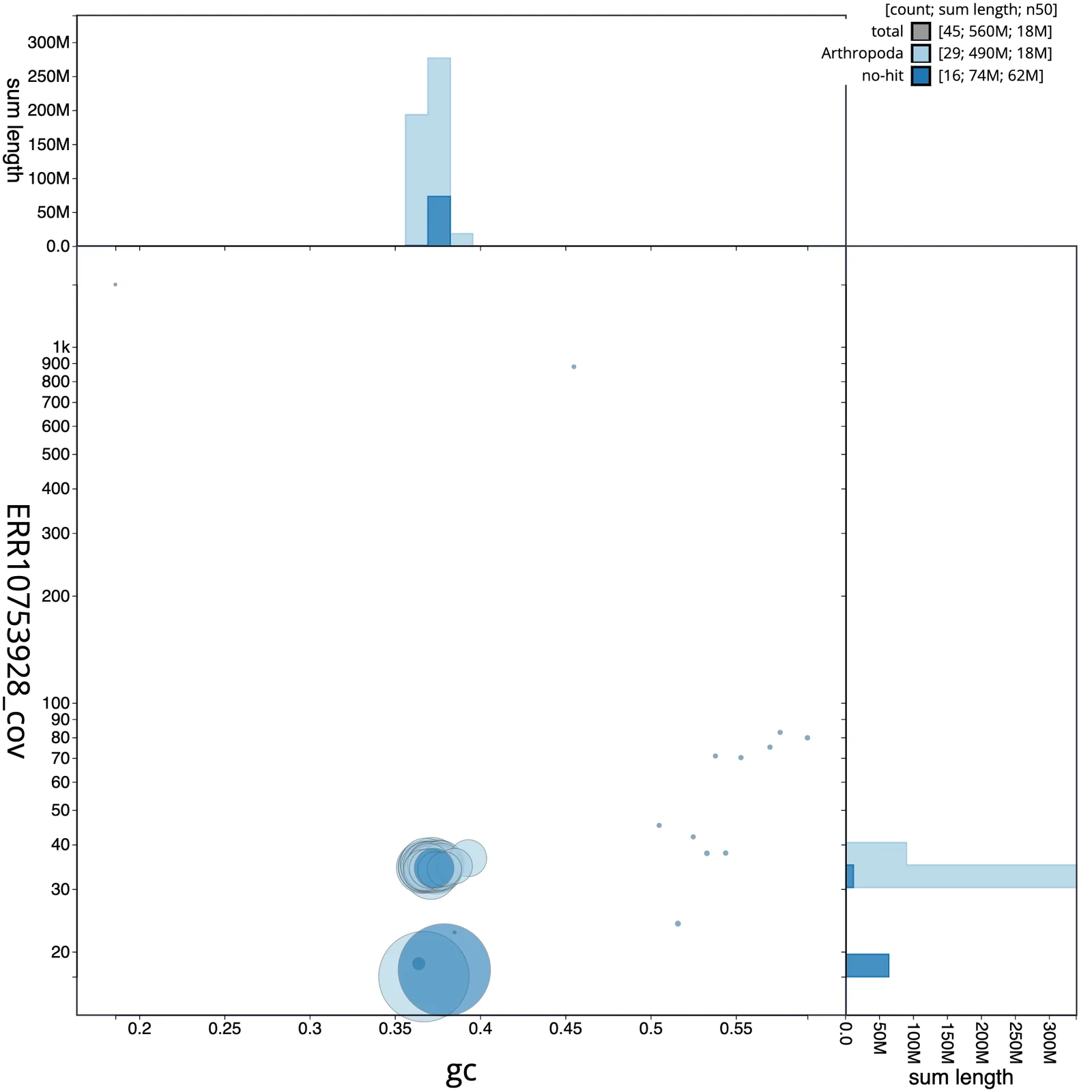
Genome assembly of *Acleris cristana*, ilAclCris2.1: BlobToolKit GC-coverage plot. Scaffolds are coloured by phylum. Circles are sized in proportion to scaffold length. Histograms show the distribution of scaffold length sum along each axis. An interactive version of this figure is available at https://blobtoolkit.genomehubs.org/view/ilAclCris2.1/dataset/CAOCPX01/blob.

**Figure 4.**
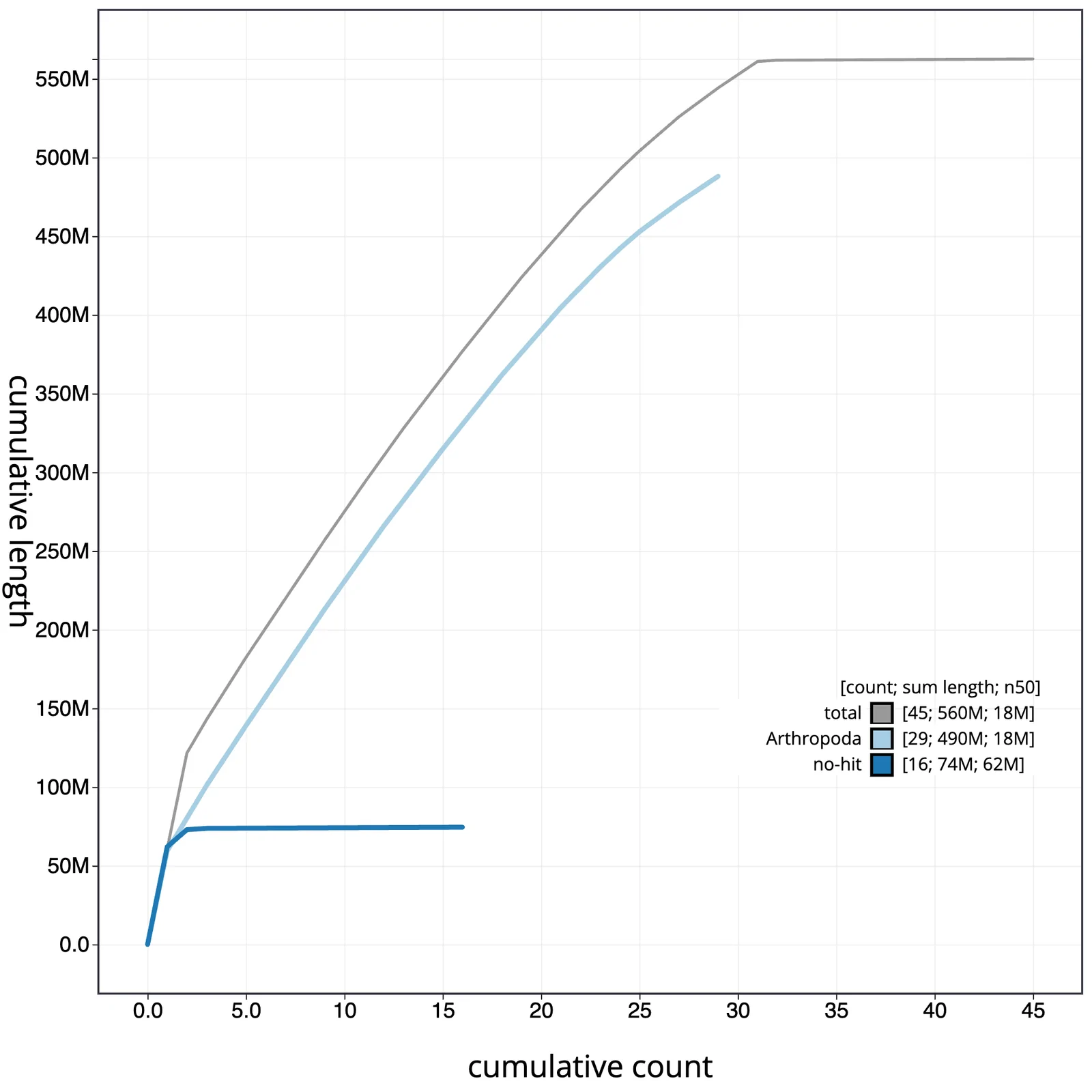
Genome assembly of *Acleris cristana*, ilAclCris2.1: BlobToolKit cumulative sequence plot. The grey line shows cumulative length for all scaffolds. Coloured lines show cumulative lengths of scaffolds assigned to each phylum using the buscogenes taxrule. An interactive version of this figure is available at https://blobtoolkit.genomehubs.org/view/ilAclCris2.1/dataset/CAOCPX01/cumulative.

**Figure 5.**
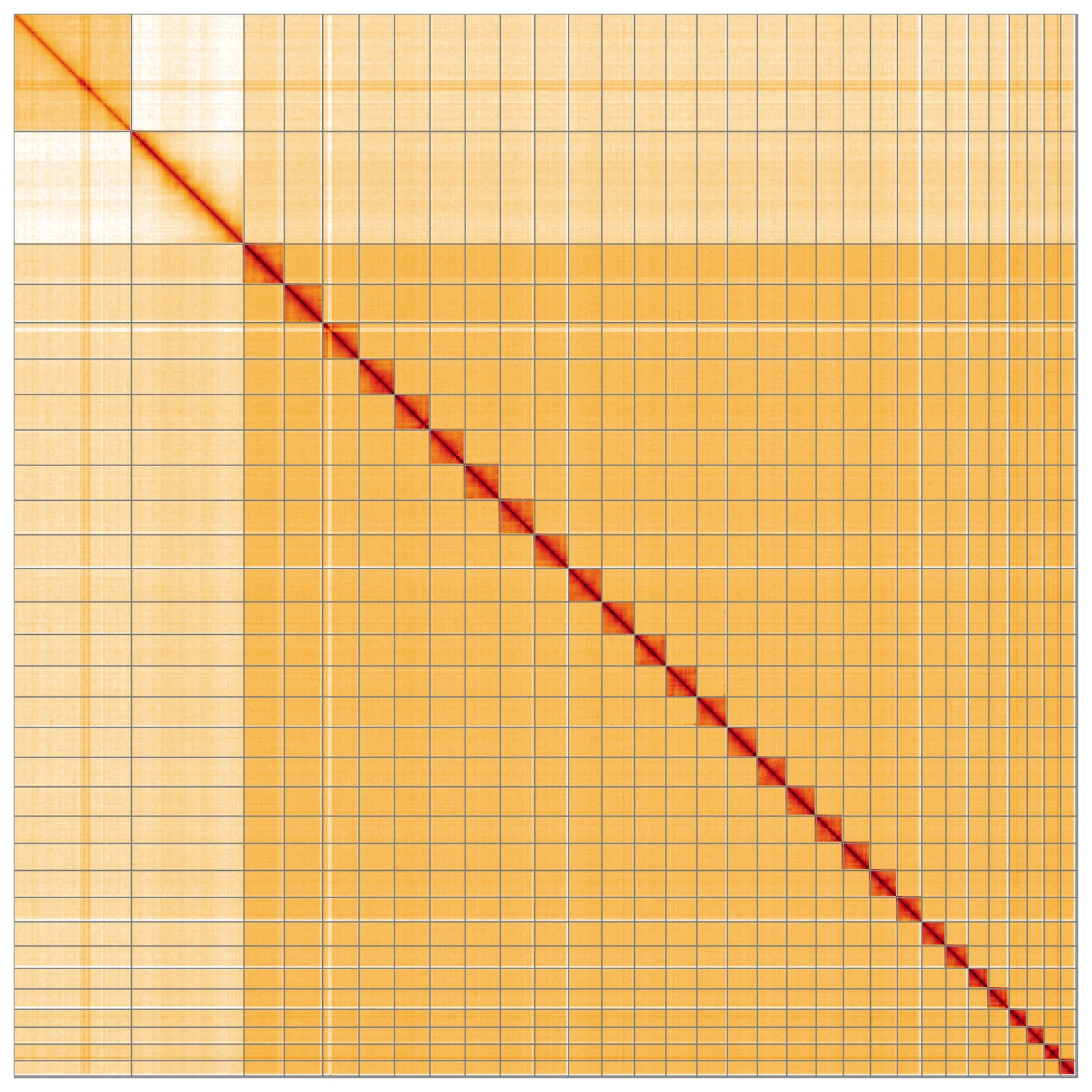
Genome assembly of *Acleris cristana*, ilAclCris2.1: Hi-C contact map of the ilAclCris2.1 assembly, visualised using HiGlass. Chromosomes are shown in order of size from left to right and top to bottom. An interactive version of this figure may be viewed at https://genome-note-higlass.tol.sanger.ac.uk/l/?d=DY0q7m1NSh2aFyNPjT93pw.

**Table 2.** Chromosomal pseudomolecules in the genome assembly of *Acleris cristana*, ilAclCris2.

INSDC accession	Name	Length (Mb)	GC%
OX411773.1	1	21.28	37.5
OX411774.1	2	20.25	37
OX411775.1	3	19.17	37
OX411776.1	4	18.76	36.5
OX411777.1	5	18.72	37.5
OX411778.1	6	18.6	36.5
OX411779.1	7	18.55	36.5
OX411780.1	8	18.19	37
OX411781.1	9	17.81	37
OX411782.1	10	17.62	37
OX411783.1	11	17.24	37
OX411784.1	12	16.61	37.5
OX411785.1	13	16.38	37
OX411786.1	14	16.05	37.5
OX411787.1	15	15.83	37.5
OX411788.1	16	15.59	37
OX411789.1	17	15.49	37.5
OX411790.1	18	14.4	37
OX411791.1	19	14.32	37
OX411792.1	20	14.26	37.5
OX411793.1	21	12.86	37.5
OX411794.1	22	12.76	37
OX411795.1	23	11.82	36.5
OX411796.1	24	10.84	37.5
OX411797.1	25	10.78	37
OX411798.1	26	9.42	39.5
OX411799.1	27	9	37.5
OX411800.1	28	8.7	38.5
OX411801.1	29	8.09	38
OX411771.1	W	62.08	38
OX411772.1	Z	59.52	36.5
OX411802.1	MT	0.02	19

The estimated Quality Value (QV) of the final assembly is 65.9 with *k*-mer completeness of 100%, and the assembly has a BUSCO v5.3.2 completeness of 98.3% (single 97.6% duplicated = 0.8%), using the lepidoptera_odb10 reference set ( *n* = 5,286).

Metadata for specimens, spectral estimates, sequencing runs, contaminants and pre-curation assembly statistics can be found at https://links.tol.sanger.ac.uk/species/758705.

## Genome annotation report

The *A. cristana* genome assembly (GCA_948252455.1) was annotated using the Ensembl rapid annotation pipeline ( Table 1; https://rapid.ensembl.org/Acleris_cristana_GCA_948252455.1/Info/Index). The resulting annotation includes 22,144 transcribed mRNAs from 12,598 protein-coding and 1,631 non-coding genes.

## Methods

### Sample acquisition and nucleic acid extraction

Two *Acleris cristana* specimens (specimen number Ox000993 and Ox000832, individuals ilAclCris2 and ilAclCris1) were collected from Wytham Woods, Oxfordshire (biological vice-county Berkshire), UK (latitude 51.77, longitude –1.34) on 2020-11-21 and 2020-08-01 respectively. The specimens were taken from the woodland habitat by Douglas Boyes (University of Oxford) using a light trap. The specimens were identified by the collector and snap-frozen on dry ice.

The sample was prepared for DNA extraction in the Tree of Life laboratory, Wellcome Sanger Institute (WSI). The ilAclCris2 specimen was weighed and dissected on dry ice. Whole organism tissue was disrupted using a Nippi Powermasher fitted with a BioMasher pestle. DNA was extracted at the WSI Scientific Operations core using the Qiagen MagAttract HMW DNA kit, according to the manufacturer’s instructions.

### Sequencing

Pacific Biosciences HiFi circular consensus DNA sequencing libraries were constructed according to the manufacturers’ instructions. DNA sequencing was performed by the Scientific Operations core at the WSI on Pacific Biosciences SEQUEL II (HiFi) instrument. Hi-C data were also generated from whole organism tissue of ilAclCris1 using the Arima2 kit and sequenced on the Illumina NovaSeq 6000 instrument.

### Genome assembly, curation and evaluation

Assembly was carried out with Hifiasm ( Cheng *et al.*, 2021) and haplotypic duplication was identified and removed with purge_dups ( Guan *et al.*, 2020). The assembly was then scaffolded with Hi-C data ( Rao *et al.*, 2014) using YaHS ( Zhou *et al.*, 2023). The assembly was checked for contamination as described previously ( Howe *et al.*, 2021). Manual curation was performed using HiGlass ( Kerpedjiev *et al.*, 2018) and Pretext ( Harry, 2022). The mitochondrial genome was assembled using MitoHiFi ( Uliano-Silva *et al.*, 2022), which runs MitoFinder ( Allio *et al.*, 2020) or MITOS ( Bernt *et al.*, 2013) and uses these annotations to select the final mitochondrial contig and to ensure the general quality of the sequence.

A Hi-C map for the final assembly was produced using bwa-mem2 ( Vasimuddin *et al.*, 2019) in the Cooler file format ( Abdennur & Mirny, 2020). To assess the assembly metrics, the *k*-mer completeness and QV consensus quality values were calculated in Merqury ( Rhie *et al.*, 2020). This work was done using Nextflow ( Di Tommaso *et al.*, 2017) DSL2 pipelines “sanger-tol/readmapping” ( Surana *et al.,* 2023a) and “sanger-tol/genomenote” ( Surana *et al.*, 2023b). The genome was analysed within the BlobToolKit environment ( Challis *et al.*, 2020) and BUSCO scores ( Manni *et al.*, 2021; Simão *et al.*, 2015) were calculated.

Table 3 contains a list of relevant software tool versions and sources.

**Table 3.** Software tools: versions and sources.

Software tool	Version	Source
BlobToolKit	4.0.7	https://github.com/blobtoolkit/blobtoolkit
BUSCO	5.3.2	https://gitlab.com/ezlab/busco
Hifiasm	0.16.1-r375	https://github.com/chhylp123/hifiasm
HiGlass	1.11.6	https://github.com/higlass/higlass
Merqury	MerquryFK	https://github.com/thegenemyers/MERQURY.FK
MitoHiFi	2	https://github.com/marcelauliano/MitoHiFi
PretextView	0.2	https://github.com/wtsi-hpag/PretextView
purge_dups	1.2.3	https://github.com/dfguan/purge_dups
sanger-tol/genomenote	v1.0	https://github.com/sanger-tol/genomenote
sanger-tol/readmapping	1.1.0	https://github.com/sanger-tol/readmapping/tree/1.1.0
YaHS	1.2a	https://github.com/c-zhou/yahs

### Genome annotation

The Ensembl gene annotation system ( Aken *et al.*, 2016) was used to generate annotation for the *Acleris cristana* assembly (GCA_948252455.1). Annotation was created primarily through alignment of transcriptomic data to the genome, with gap filling via protein-to-genome alignments of a select set of proteins from UniProt ( UniProt Consortium, 2019).

### Wellcome Sanger Institute – Legal and Governance

The materials that have contributed to this genome note have been supplied by a Darwin Tree of Life Partner. The submission of materials by a Darwin Tree of Life Partner is subject to the **‘Darwin Tree of Life Project Sampling Code of Practice’**, which can be found in full on the Darwin Tree of Life website here. By agreeing with and signing up to the Sampling Code of Practice, the Darwin Tree of Life Partner agrees they will meet the legal and ethical requirements and standards set out within this document in respect of all samples acquired for, and supplied to, the Darwin Tree of Life Project.

Further, the Wellcome Sanger Institute employs a process whereby due diligence is carried out proportionate to the nature of the materials themselves, and the circumstances under which they have been/are to be collected and provided for use. The purpose of this is to address and mitigate any potential legal and/or ethical implications of receipt and use of the materials as part of the research project, and to ensure that in doing so we align with best practice wherever possible.

The overarching areas of consideration are:

- Ethical review of provenance and sourcing of the material
- Legality of collection, transfer and use (national and international).

Each transfer of samples is further undertaken according to a Research Collaboration Agreement or Material Transfer Agreement entered into by the Darwin Tree of Life Partner, Genome Research Limited (operating as the Wellcome Sanger Institute), and in some circumstances other Darwin Tree of Life collaborators.

## Data Availability

European Nucleotide Archive: *Acleris cristana* (tufted button). Accession number PRJEB58659; https://identifiers.org/ena.embl/PRJEB58659. ( Wellcome Sanger Institute, 2023) The genome sequence is released openly for reuse. The *Acleris cristana* genome sequencing initiative is part of the Darwin Tree of Life (DToL) project. All raw sequence data and the assembly have been deposited in INSDC databases. Raw data and assembly accession identifiers are reported in Table 1.

## Author information

Members of the University of Oxford and Wytham Woods Genome Acquisition Lab are listed here: https://doi.org/10.5281/zenodo.4789928.

Members of the Darwin Tree of Life Barcoding collective are listed here: https://doi.org/10.5281/zenodo.4893703.

Members of the Wellcome Sanger Institute Tree of Life programme are listed here: https://doi.org/10.5281/zenodo.4783585.

Members of Wellcome Sanger Institute Scientific Operations: DNA Pipelines collective are listed here: https://doi.org/10.5281/zenodo.4790455.

Members of the Tree of Life Core Informatics collective are listed here: https://doi.org/10.5281/zenodo.5013541.

Members of the Darwin Tree of Life Consortium are listed here: https://doi.org/10.5281/zenodo.4783558.
