# Supplementary material for: The genome sequence of the Tufted Button, Acleris cristana (Denis & Schiffermüller, 1775)
Source: Wellcome Open Res. 2026 Aug 27;8:236. Originally published 2023 Jun 7. [Version 2] doi: 10.12688/wellcomeopenres.19508.2 (PMC13562962; doi:10.12688/wellcomeopenres.19508.2)
Supplement: Peer review history

# Reviewer report: Reviewer response for version 2

**DOI:** 10.21956/wellcomeopenres.30151.r165884

**Published:** 2026-09-02

**Authors:** Yannis Schoeneberg

**Affiliations:** 1 Trier University, Tier, Germany

In their revision, the authors have significantly improved the Methods section and addressed all of my previously raised concerns. I would like to commend the authors on the outstanding quality of their genome assembly, which will provide a valuable resource for future studies.

 I have no further comments.

Are sufficient details of methods and materials provided to allow replication by others?

No

Is the rationale for creating the dataset(s) clearly described?

Yes

Are the datasets clearly presented in a useable and accessible format?

Yes

Are the protocols appropriate and is the work technically sound?

Yes

Reviewer Expertise:

genome assembly; comparative genomics; phylogenomics

I confirm that I have read this submission and believe that I have an appropriate level of expertise to confirm that it is of an acceptable scientific standard.

# Reviewer report: Reviewer response for version 1

**DOI:** 10.21956/wellcomeopenres.21610.r82252

**Published:** 2024-05-13

**Authors:** Yannis Schoeneberg

**Affiliations:** 1 Trier University, Tier, Germany

In this article the authors describe how they sequenced and assembled the genome of Acleris cristana, a moth of the family Torticidae. The genome was assembled to chromosome level, annotated and the sex chromosomes were identified.

 The authors assembled a high quality genome assembly, which will be an important resource for the study of this species and beyond. The assembly strategy is appropriate, and the quality metrics indicate an assembly of outstanding quality. However, I found the methods to be unreproducible, as the section contained too little detail and important information was absent. Apart from that, I only have minor suggestions and would like to congratulate the authors on their genome assembly of exceptional quality.

 Best whishes

 Yannis Schöneberg

 Are sufficient details of methods and materials provided to allow replication by others?

 No - The method section seems to be incomplete and often lacks detail necessary to make this publication reproducible. I advise the authors to thoroughly check that all steps were included in the methods (e.g. BlobtoolKit lacks a description how the input data was generated, the identification of sex chromosomes is missing entirely). Furthermore, a very brief explanation why each step was performed would greatly enhance the understandability for readers not being experts in genome assembly.

 # Detailed Comments:

 ## Background:

   - I think it would be necessary to cite a reference for the statement that there are several genes influencing the colours of individual pattern elements.

 ## Genome sequence report:

   - I assume it is the case, but it would be nice for the reader if the authors specifically mention that the number of chromosome-level scaffolds matches the expected karyotype.

   - I'm not sure what is meant with "not fully phased". This should be described in more detail.

 ## Genome annotation report

   - The rapid annotation pipeline seems to be a standardized pipeline. However, that doesn't guarantee it generates reliable annotations. I haven't tried this pipeline, but to my experience many standardized pipelines (e.g. NCBI RefSeq) perform poorly on arthropod genomes, as they are usually designed for vertebrates. I suggest providing the reader with quality metrics for the annotation, such as BUSCO score, or Annotation Edit Distance (AED), or else. Otherwise, the annotation's quality is unclear.

 ## Methods:

 In general, I think the methods should have more detail and the authors should thoroughly check that each step is properly described (e.g. sex chromosome identification is missing). I think that short explanations why a certain step was conducted could help the reader understand the workflow. As a couple of programs and work steps were missing from the text and table 3, I advise the authors to check whether all relevant programs and work steps are listed.

 ## Genome assembly, curation and evaluation:

   - Hifiasm can be run with HIFI data alone or combined with the Hi-C data. It would be good to briefly mention which data was used.

   - This might be nit picking, but purge_dups is also part of the Hifiasm workflow. For clarity, I think it should be mentioned whether it was run as part of Hifiasm, standalone, or both.

   - As much as I understand, Pretext is not a single program but a prefix used for several programs (e.g. PretextView or PretextMap). These programs seem to be bundled in the PretextSuite, but for clarity I think it would be best to mention and cite each program individually and briefly describe what it was used for.

   - A precise description of how the HiC contact map was generated is missing. As I'm not aware that bwa-mem2 outputs cooler files, I assume the authors used the cooler program suite to generate these files, and the contact maps. If this is the case, the program is also missing from table 3.

   - For the mitochondrial genome assembly it should be specifically stated whether MitoFinder or MITOS was used.

   - BlobtoolKit requires mapped reads and a taxonomic annotation of the scaffolds as input files. The information which programs and data were used to generate the input files is missing from the text and table 3.

   - It was mentioned in the genome sequence report, but the BUSCO set used for QC of the assembly should be named again in the methods.

   - The MerquryFK workflow is different and has more possibilities than the original Merqury workflow. Please describe exactly what was done and name and cite the programs utilized (e.g. FastK).

   - The methods lack a description of how the sex chromosomes were identified.

 ## Table 3:

   - Depending on what was used for the mitochondrial assembly, MitoFinder or MITOS is missing from the table.

   - For Merqury, I would suggest to call it MerquryFK in the Software tool column and the text, otherwise there might be confusion. I assume the version used is v1.0.0 as this is the tag of the only release on the Github repository.

   - bwa-mem2 is missing from the table.

   - Nextflow is missing from the table.

   - Ensembl Annotation Pipeline is missing from the table.

   - As much as I understand the author's workflow, they used several of the programs bundled in the Pretext-suite, not just PretextView. Therefore, I think it would be best to cite each of the programs individually.

 ## Genome annotation

   - I think this section should generally be described in more detail. I suggest to include a very short description of all work steps and citation of the programs utilized by the pipeline.

   - It's unclear which training/reference data was used for the annotation. I suggest giving accessions for the transcriptomic and protein dataset if possible.

Are sufficient details of methods and materials provided to allow replication by others?

No

Is the rationale for creating the dataset(s) clearly described?

Yes

Are the datasets clearly presented in a useable and accessible format?

Yes

Are the protocols appropriate and is the work technically sound?

Yes

Reviewer Expertise:

genome assembly; comparative genomics; phylogenomics

I confirm that I have read this submission and believe that I have an appropriate level of expertise to confirm that it is of an acceptable scientific standard, however I have significant reservations, as outlined above.

# Reviewer report: Reviewer response for version 1

**DOI:** 10.21956/wellcomeopenres.21610.r67857

**Published:** 2023-10-10

**Authors:** Feng Zhang

**Affiliations:** 1 Department of Entomology, College of Plant Protection, Nanjing Agricultural University, Nanjing, China

The authors describe the first genome assembly of the Tufted Button *Acleris cristana*, a common but poorly genetic studied moth distributes in Europe. Combining HiFi long reads and Hi-C data from an adult female moth, the authors established a chromosomal-level assembly of ~562.6Mb. The genome contains 29 autosomes and two sex chromosomes (Z and W). Several metrics suggest that genome has a high completeness and contiguity.

 There is a discrepancy in the paper as to whether this represents a single or two female samples (line 1 in Methods says two females were collected, the Genome sequence report says one female for sequencing). The authors need to clarify the reason why they only used a single female instead of two.

 In Genome sequence report, the authors mentioned W and Z chromosomes are large without giving size of sex chromosomes. It could be clearer if the authors provide size information for W and Z chromosomes to explain why they can be classified as “large chromosome”.

 Minor revisions:

1. In Background, delete “the” in front of “family Tortricidae”.
2. In Background, change “The species” to “This species”.
3. In Methods, add the name of “collector”.

Reviewer Expertise:

Insect genomics, Phylogenomics

I confirm that I have read this submission and believe that I have an appropriate level of expertise to confirm that it is of an acceptable scientific standard.
